# 30-Year Survival After the Fontan Operation in Denmark

**DOI:** 10.1007/s00246-025-03896-4

**Published:** 2025-05-22

**Authors:** Benjamin Kelly, Maren Ravndal, Anne Kathrine Møller Nielsen, Emil Krogh, Vibeke E. Hjortdal, Lars Idorn

**Affiliations:** 1 Department of Cardiothoracic Surgery, Copenhagen University Hospital, Blegdamsvej 9, 2100 Copenhagen, Denmark; 2 Department of Clinical Medicine, Aarhus University, Aarhus, Denmark; 3 Department of Pediatrics, Copenhagen University Hospital, Copenhagen, Denmark

**Keywords:** Single ventricle, Fontan, Hypoplastic left heart syndrome, Long-term survival

## Abstract

This study aimed to evaluate the nationwide 30-year survival among Danish univentricular heart patients who underwent Fontan completion between 1977 and 2023. Secondary objectives included assessment of the impact of era, Fontan type, and ventricular dominance on survival. Finally, clinical performance and the prevalence of select morbidity were described for survivors. The Danish Fontan cohort of 301 patients was identified using surgical registries and digitalized health records. Information on mortality, morbidity, and clinical performance was collected from digital medical records using national personal identification numbers. Survival analysis was conducted using Kaplan–Meier estimates, and morbidity outcomes were compared for three consecutive age groups (< 18 years, 18–29 years, and ≥ 30 years). The 30-year survival rate after Fontan completion was 87%, while freedom from death, transplant, or Fontan takedown was 79%. Improved outcomes were observed for patients undergoing Fontan completion after 1997 and those with a non-hypoplastic left heart syndrome (HLHS) diagnosis. Morbidity, including ventricular dysfunction, arrhythmia, significant atrioventricular valve regurgitation, and protein-losing enteropathy, was present in 38% of survivors and increased with age. The proportion of (self-reported) physically active individuals and their %pred VO2peak declined with age, with those aged ≥ 30 years demonstrating the highest prevalence of Fontan-related complications (56%) and the lowest exercise capacity (51%pred VO2peak). In conclusion, long-term survival after Fontan completion was 87%. Survival has improved in the later era and was superior for those with a non-HLHS diagnosis. The prevalence of select morbidity was substantial at 38% while also displaying an age-related increase.

**Supplementary Information:**

The online version contains supplementary material available at 10.1007/s00246-025-03896-4.

## Introduction

The Fontan procedure was pioneered more than 50 years ago [1, 2]. Since then, both the surgical procedure itself and the staging leading up to the final circulation have undergone continuous refinement [3–5]. Patient selection, perioperative and intensive care management, and follow-up care have also all changed, progressed, and improved over the years [5, 6]. The impact of these early improvements has translated into increased survival in more recent times; however, the improved survival seems predominantly confined to the first postoperative year following Fontan completion [7, 8]. Although some studies point toward a 25-year postoperative survival estimate exceeding 80% [9], the long-term survival with an intact Fontan circulation remains largely unknown, especially for those with lateral-type or extracardiac conduit-type Fontan. While increased survival is celebrated, improved survival comes at the expense of prolonged exposure to a notably different circulation. This exposure is known to be inherently associated with significant late complications [10–13]. In the current study, we aimed to provide elaborate data on 30-year survival with an intact Fontan circulation for individuals undergoing Fontan completion in Denmark from 1977 to 2023. Secondary aims included reporting long-term outcomes for specific subgroups, including the surgical type of Fontan and the time period of operation, and finally providing an overview of select morbidity prevalent in the surviving population.

## Methods

### Patient Inclusion

The Danish Fontan cohort includes all Danish citizens born with a single ventricle congenital heart defect and palliated with a Fontan circulation between January 1 st, 1977, and December 31 st, 2023. Early surgical registries, digitalized health records based on a national personal identification number, and participation in previous studies [14, 15] allowed for both nationwide identification, high-quality survival data, and linkage with in-hospital medical records. Additional patients identified through outpatient clinics, who were operated outside of Denmark, were also included in the cohort. The study received approval from the National Scientific Ethics Committee (H-20028226) and was conducted in accordance with the Helsinki Declaration. The need for informed consent was waived due to the study's retrospective nature.

### Survival Analysis

Survival with intact Fontan circulation was assessed from the completion date to the composite endpoint of death, heart transplantation, or Fontan takedown. Patients alive with intact Fontan circulation at the study’s end (December 31, 2023) were censored, as were emigrants at the time of their last known Danish hospital contact. Survival comparisons were made based on key parameters, including Fontan completion before or after 1997, Fontan type, and hypoplastic left heart syndrome. The 1997 cutoff reflects the introduction of extracardiac conduit procedures and other advances, and is consistent with the eras of a prior study [7].

### Clinical Outcomes

Medical records provided data on demographics, ventricular morphology, surgical history, and post-Fontan outcomes, including heart transplantation or takedown. Clinical performance and complications (e.g., arrhythmia, protein-losing enteropathy, and post-Fontan surgeries) were extracted from 2021 to 2023 outpatient records. Ejection fraction and atrioventricular valve regurgitation were based on latest cMRI or in the cases where no cMRI was available latest echocardiography. An ejection fraction < 40% was defined as moderate-to-severely reduced, and a regurgitation fraction > 25% was defined as moderate-to-severe regurgitation. If no cMRI had been performed, the latest echo report was used, and in case of ambiguity, the echo images were reviewed by an ACHD cardiologist or pediatric cardiologist, and EF and valve regurgitation assessed by eye-balling. Information on exercise capacity was collected from the most recent cardiopulmonary exercise test. These were performed using an upright bicycle ergometer (protocol: Ramp or incremental step), and the %predicted VO2peak (for patients aged > 10 years) was calculated using the equation described by Mylius et al. [16]. Self-evaluated level of physical activity was categorized based on the previously collected age-appropriate questionnaires (PAQ-A, PAQ-C, or GPAQ). The methods for exercise capacity and self-evaluated level of physical activity have been described in detail elsewhere [17]. Clinical outcomes were stratified into three age groups (< 18 years, 18–29 years, ≥ 30 years), excluding those who reached the composite endpoint.

### Statistics

Continuous data were reported as mean ± SD or median (IQR) for normally and non-normally distributed data, respectively. Group comparisons used Student’s *t* test, Wilcoxon rank-sum test, chi-squared, or Fisher’s exact test as appropriate. Kaplan–Meier curves estimated survival probabilities, with log-rank tests comparing distributions. Analyses were conducted using R (v4.2.2), with statistical significance set at *p* < 0.05.

## Results

**Fig. 1:**
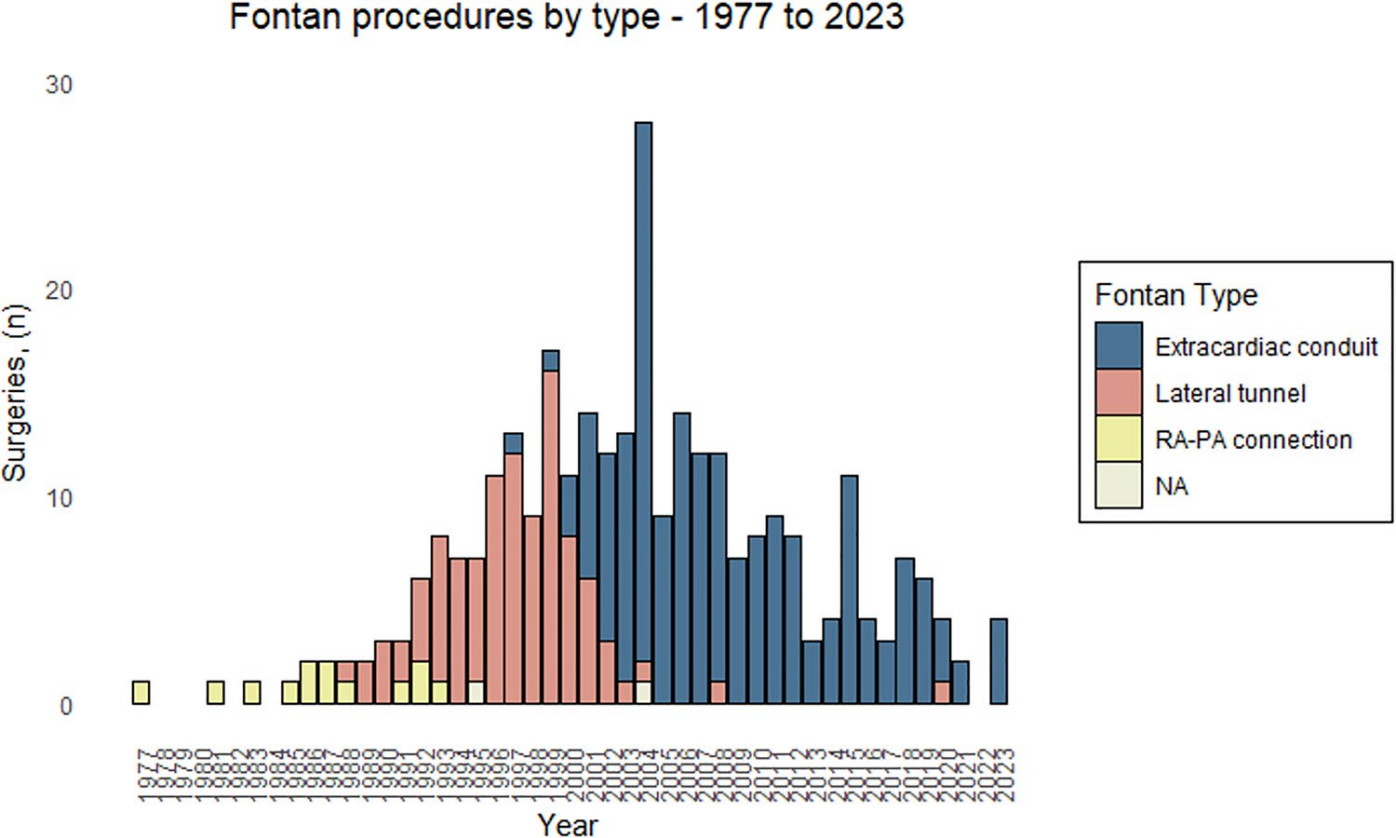
Histogram showing the year of operation along with the type of Fontan performed. For the three individuals operated abroad, year of Fontan operation and type of operation are added if the information was available. During the period from 1977 to 2023, 13 (4%) received an atriopulmonary Fontan, 100 (33%) received a lateral tunnel-type Fontan, while 186 (62%) received an extracardiac conduit-type Fontan. The type of operation was unknown for 3 (1%)

From January 1, 1977, to December 31, 2023, a total of 301 Danish patients underwent a Fontan procedure. For demographic and surgical characteristics, see Fig. 1 and Table 1.

**Table 1:** Demographical and surgical characteristics

	All (*n* = 301)	Era 1977–1996 (*n* = 57)	Era 1997–2023 (*n* = 244)	*p* value
*Sex*				
Female	117 (39%)	24 (42%)	93 (38%)	–
Male	184 (61%)	33 (58%)	151 (62%)	
*Morphology*				
Left ventricle	188 (63%)	43 (75%)	145 (59%)	0.04
Right ventricle	113 (38%)	14 (25%)	99 (41%)	
*Diagnosis*				
Tricuspid atresia	72 (24%)	23 (40%)	49 (20%)	–
Hypoplastic left heart syndrome	67 (22%)	9 (16%)	58 (24%)	
Double inlet left ventricle	61 (20%)	11 (19%)	50 (20%)	
Unbalanced atrioventricular septal defect	32 (11%)	3 (5%)	29 (12%)	
Pulmonary atresia	31 (10%)	5 (9%)	26 (11%)	
Other	38 (13%)	6 (11%)	32 (13%)	
Bidirectional Glenn before Fontan	234 (78%)	11 (19%)	223 (91%)	< 0.001
*Fontan procedure*				
Atriopulmonal (classical Fontan)	13 (4%)	13 (23%)	0 (0%)	< 0.001
Lateral tunnel	100 (33%)	43 (75%)	58 (23%)	
Extracardiac conduit	186 (62%)	0 (0%)	185 (76%)	
*Missing*	3 (1%)	1 (2%)	2 (1%)	
Age at Fontan completion, years	3.5 (2.7–5.3)	4.4 (3.1–6.2)	3.2 (2.7–4.8)	< 0.01

Data are presented as *n*(%) or median (IQR)

**Fig. 2:**
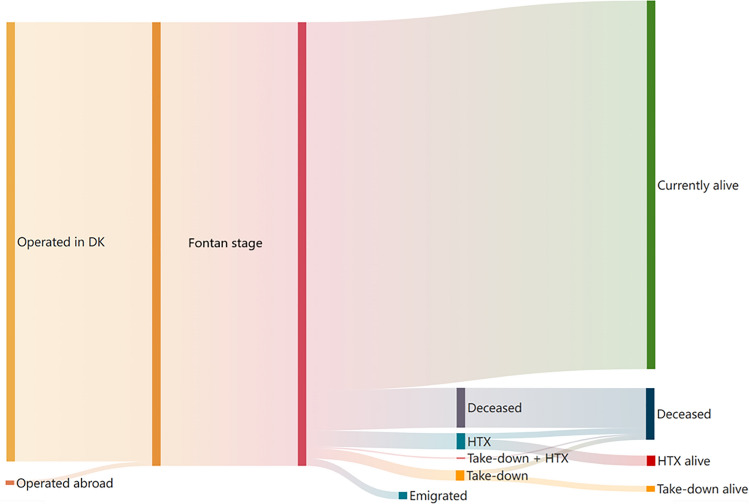
Sankey chart displaying the course of the Danish Fontan population with regard to survival, takedown, transplant, and death. 298 individuals born with a univentricular heart underwent Fontan completion in Denmark

At the end of the follow-up period, 250 individuals were still alive with a Fontan circulation, 35 individuals (12%) were deceased, 7 remained alive after heart transplantation, and 4 were alive with a bidirectional Glenn circulation after Fontan takedown and were not considered candidates for reattempted Fontan completion. Among the deceased, 14 individuals (40%) died within the first year after Fontan completion. See Fig. 2. The median times from Fontan completion to death or heart transplantation were 2.8 years (IQR 0.1–15.8) and 4.6 years (IQR 1.2–13.4) respectively, and the median time from Fontan procedure to takedown was 16.9 days (IQR 1.9–39.4). The median follow-up time for overall survival and survival with an intact Fontan circulation was 18.3 years and 17.2 years respectively.

**Fig. 3:**
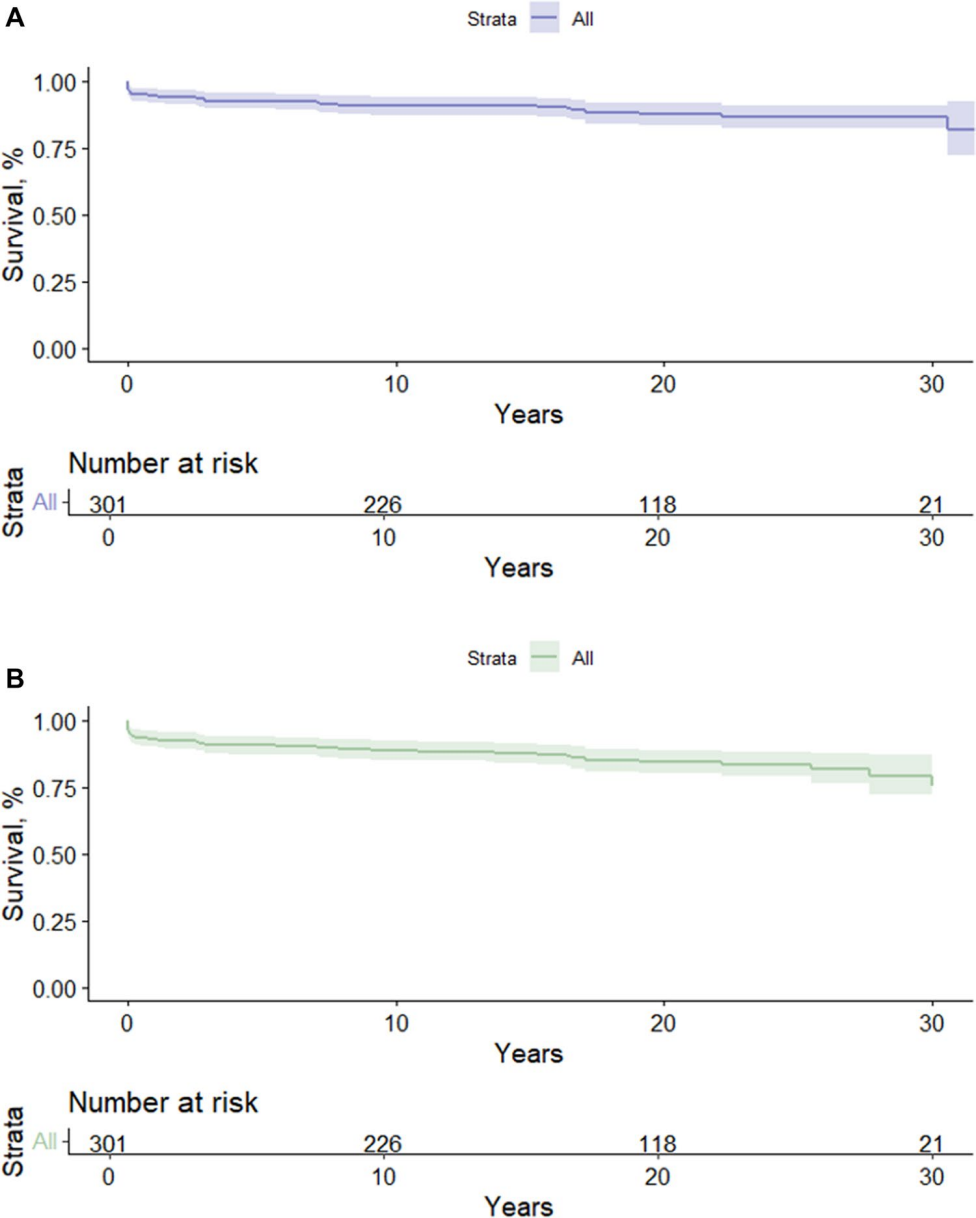
**A** Overall survival in years following Fontan completion. The 5, 15, and 30-year survival rates were 0.93 (95% CI 0.90–0.96), 0.91 (95% CI 0.87–0.94), and 0.87 (95% CI 0.82–0.91). **B** Event-free survival with an intact Fontan circulation. The 5, 15, and 30-year survival rates with an intact Fontan circulation were 0.91 (95% CI 0.87–0.94), 0.88 (95% CI 0.84–0.91), and 0.79 (95% CI 0.72–0.87)

The overall survival following Fontan completion was 93% (95% CI 90–96) at 5 years and 87% (95% CI 82–91) at 30 years. Survival with an intact Fontan circulation was 91% (95% CI 87–94) at 5 years and 79% (95% CI 72–87) at 30 years. See Fig. 3.

To compare individuals operated in an early versus a late era, patients were divided into two groups: Those with Fontan completion in the period 1977–1996 (*n* = 57) and those with completion in the period 1997–2023 (*n* = 244). When comparing distributions and characteristics between the two groups, the Fontan procedures completed in the late era were younger at Fontan completion, had a greater proportion of individuals with systemic right ventricle, more were staged with a bidirectional Glenn, and the extracardiac conduit became the preferred operation of choice. See Table 1.

**Fig. 4:**
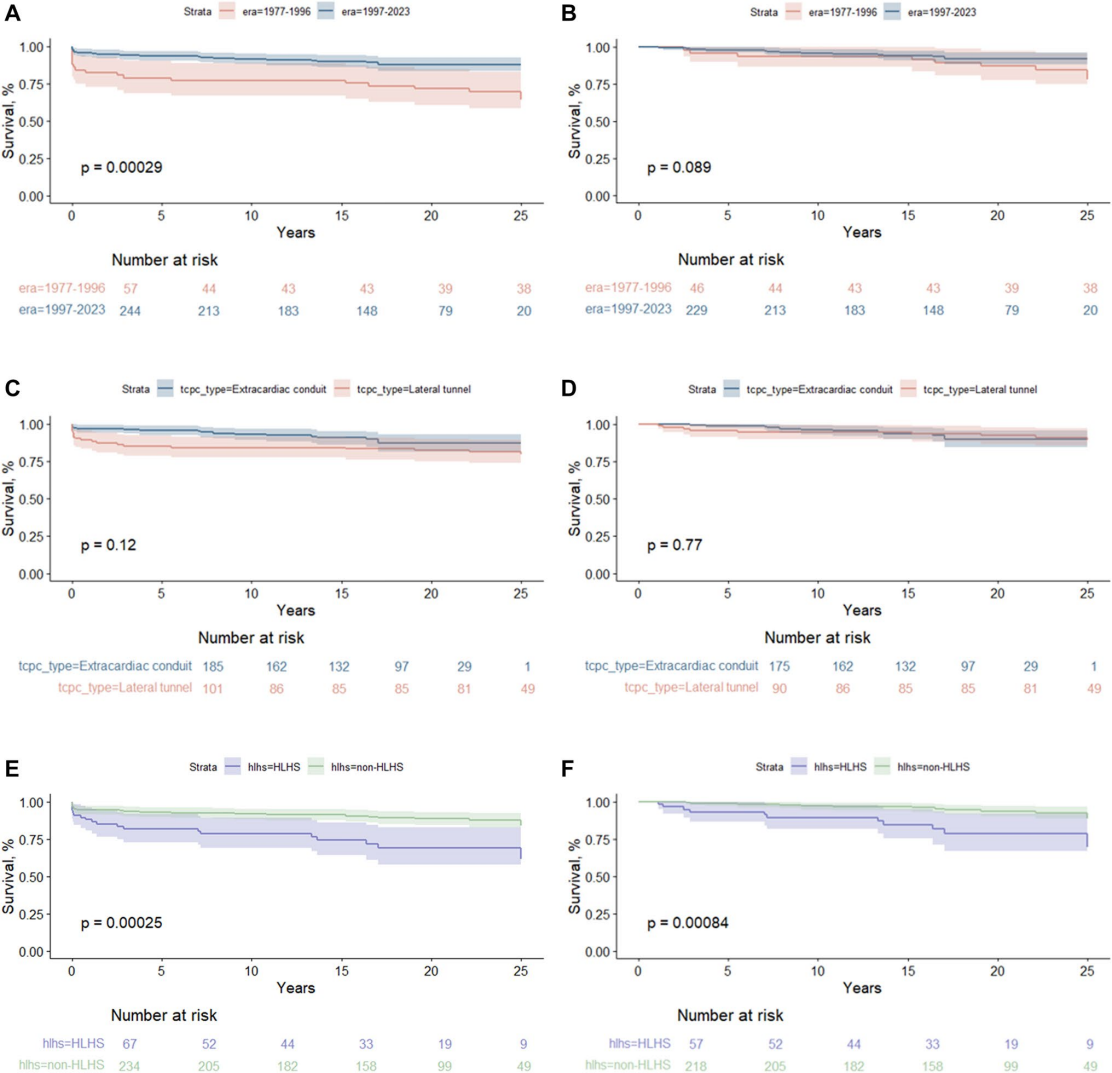
Event-free survival following Fontan completion (left column) and event-free survival conditional of surviving the first year after the Fontan operation (right column). Overall, being operated in the early era (a) or a diagnosis of hypoplastic left heart syndrome (g) displayed reduced event-free survival. Only being born with a hypoplastic left heart remained significantly associated with reduced survival when making event-free survival conditional of surviving the first year after Fontan completion. Survival and 95% CI for all survival curves are available in the supplementary

Event-free survival with intact Fontan circulation improved significantly between eras. For those operated in the early period, the event-free survival was 79% (95% CI 69–90) at 5 years and 64% (95% CI 53–78) at 25 years compared to 93% (95% CI 90–97) and 88% (95% CI 83–92) in the late period. The improvement was primarily due to better first-year survival in the late era. Survival by surgical type showed similar trends. Event-free survival for lateral tunnel Fontan was 85% (95% CI 78–92) and 79% (95% CI 72–88) at 5 and 25 years, respectively. For extracardiac conduit Fontan, rates were 96% (95% CI 93–99) and 87% (95% CI 81–93). Conditional late event-free survival at 25 years was comparable: 89% (95% CI 83–96) for lateral tunnel and 90% (95% CI 85–96) for extracardiac conduit. Finally, individuals with hypoplastic left heart syndrome had lower survival rates: 82% (95% CI 73–92) and 61% (95% CI 46–82) compared to 93% (95% CI 90–96) and 84% (95% CI 77–91) for other diagnoses at 5 and 25 years, respectively. The difference was maintained for conditional survival. See Fig. 4.

At the end of inclusion, 250 individuals were alive with a Fontan circulation in Denmark. One patient had opted out of clinical follow-up and was excluded from the analysis. The mean age was 24 years ± 10, and follow-up time since Fontan completion was 19 years ± 8. Of the entire cohort, the accumulated prevalence of ventricular dysfunction, atrioventricular valve regurgitation, arrhythmia, and protein-losing enteropathy was 94 individuals (38%). See Table 2.

**Table 2:** Characteristics, morbidity, and performance by age group

	All (*n* = 249)	< 18 years (*n* = 61)	18–29 years (*n* = 125)	≥ 30 years (*n* = 63)	*p* value
*Demographics*					
Sex, Female	92 (37%)	19 (31%)	43 (34%)	30 (48%)	00.11
Age, mean ± SD	24 ± 10	12 ± 4	24 ± 3	37 ± 6	–
*Diagnosis*					
Tricuspid atresia	60 (24%)	12 (20%)	30 (24%)	18 (29%)	00.11
Hypoplastic left heart syndrome (including mitral atresia)	47 (19%)	16 (26%)	24 (19%)	7 (11%)	
Double inlet left ventricle	54 (22%)	8 (13%)	26 (21%)	20 (32%)	
Atrioventricular septal defect	27 (11%)	7 (12%)	16 (13%)	4 (6%)	
Pulmonary atresia	26 (10%)	8 (13%)	15 (12%)	3 (5%)	
Other	35 (14%)	10 (16%)	14 (11%)	11 (17%)	
*Morphology*					
Systemic left ventricle	161 (65%)	35 (57%)	78 (62%)	48 (76%)	00.07
Systemic right ventricle	88 (35%)	26 (43%)	47 (38%)	15 (24%)	
*Operative characteristics*					
Age at Fontan, median (IQR)	3 (3–5)	3 (3–4)	3 (3–4)	6 (4–12)	< 0.001
Glenn completion before Fontan	203 (82%)	59 (97%)	115 (92%)	29 (46%)	< 0.001
Extracardiac conduit	165 (66%)	61 (100%)	88 (70%)	16 (25%)	< 0.001
Lateral tunnel	79 (32%)	0 (0%)	38 (30%)	42 (67%)	
RA–PA connection	5 (2%)	0 (0%)	0 (0%)	5 (8%)	
Fenestration created	149 (60%)	35 (57%)	75 (60%)	38 (60%)	0.93
Patent at last examination	22 (9%)	9 (15%)	6 (5%)	7 (11%)	
Closed (Device or spontaneously)	127 (51%)	26 (42%)	69 (55%)	31 (49%)	
Unknown	11 (4%)	1 (2%)	7 (6%)	3 (5%)	
*Complications*					
Treated arrhythmia	62 (25%)	8 (13%)	27 (22%)	27 (43%)	< 0.001
Moderately or severely reduced ejection fraction	15 (6%)	1 (2%)	7 (6%)	7 (11%)	00.09
Moderate or severe atrioventricular valve regurgitation	25 (10%)	4 (7%)	16 (13%)	5 (8%)	00.40
Protein-losing enteropathy (PLE)	14 (6%)	1 (2%)	11 (9%)	2 (3%)	00.11
Composite endpoint morbidity	95 (38%)	11 (18%)	49 (39%)	35 (56%)	< 0.001
*Physical performance*					
%pred VO2peak^a^	56 ± 12	65 + 17	58 + 12	51 + 9	< 0.001
*Missing*	86 (35%)	42 (69%)	32 (25%)	12 (19%)	
Active^b^	64/163 (39%)	22/33 (67%)	34/89 (38%)	8/41 (20%)	< 0.001
*Missing*	86 (35%)	28 (46%)	37 (29%)	22 (35%)	
*Post-Fontan catheterizations or cardiac surgery*					
Interventional cardiac catheterization post-Fontan completion	37 (15%)	7 (11%)	20 (16%)	10 (16%)	0.047916667
Cardiac surgery post-Fontan completion	28 (11%)	2 (3%)	15 (12%)	11 (17%)	00.03

^a^Calculated using the reference equation published by Mylius et al. [17]

^b^Based on answers from either the PAQ questionnaires (individuals reporting physical activity at least once a week were classified as active) or the GPAQ questionnaire (those participating in recreational activities considered vigorous were categorized as active)

All living were stratified into three age groups: < 18 years (61 patients, 24%), 18*–*29 years (126 patients, 51%), and 30 years or older (63 patients, 25%). The younger groups living with a Fontan circulation were more likely to have been younger at Fontan completion, have received surgical staging before Fontan, and to have received an extracardiac conduit-type Fontan. Looking at the distribution of complications between the three age groups, the presence of clinically relevant arrhythmia increased with age (13.1% in patients < 18 years, 21.4% in patients 18*–*29 years, 65.5% in patients ≥ 30 years, *p* < 0.001). Similarly, the proportion of patients with any one or more of the selected complications increased in the older age groups (32.9% in patients < 18 years, 57.1% in patients 18*–*29 years, 72.6% in patients ≥ 30 years, *p* < 0.001).

Of the 249 patients, 163 (65.5%) had a cardiopulmonary exercise test (CPET) and a similar number had answered a self-evaluation questionnaire in relation to the DANFontan trial [17]. Both examinations were no more than three years old at the end of follow-up. The mean %pred VO2_peak_ was 56.6 ± 12.3% for all individuals who completed a CPET, and 39% self-reported to be active. Both the %pred VO2_peak_ and the proportion of self-reporting to be active declined with age. See Table 2 (More information about both tests is available in the supplementary).

## Discussion

In this nationwide follow-up study of the 301 individuals undergoing Fontan completion in Denmark between 1977 and 2023, we report a 30-year survival and survival with an intact Fontan circulation of 87% and 79% respectively. Fontan completion during or after 1997 or a non-HLHS cardiac diagnosis displayed superior survival, while there was no difference when comparing those who received a lateral-type Fontan with those receiving an extracardiac conduit-type Fontan. For those alive in Denmark with a Fontan circulation, the accumulated prevalence of ventricular dysfunction, atrioventricular valve regurgitation, arrhythmia, and protein-losing enteropathy increased with age, while the proportion of individuals self-reporting to be active and the percent predicted exercise capacity declined.

Postoperative survival rates in the Danish Fontan population were 87% at 30 years and survival with an intact Fontan circulation 79%. Comparing long-term outcomes after Fontan completion is complex due to variations in surgical era, procedure types, and inherent risks associated with different univentricular lesions. However, the findings compare well with other studies. For example, Nakano et al. reported 93% survival at 15 years in an all-extracardiac conduit Fontan cohort (1994–2014) from a single center [18], while Downing et al. observed 74% event-free survival at 20 years [7]. More recent studies have also explored long-term survival trends. Moon et al. reported 71% survival with an intact Fontan circulation at 25 years [19], while Poh et al. observed a 30-year death- and transplant-free survival of 75% in the bi-national Australian and New Zealand cohort [9].

When looking at the period of the Fontan operation, survival with an intact Fontan circulation improved significantly for those operated after 1997. The 25-year survival after Fontan completion improved from 64% before 1997 to 93% for those operated after 1997. This aligns with other reports of inferior survival for those operated before 1997 [7]. Pundi et al. reported a similar institutional reduction in mortality, with the 20-year survival improving from 57% (1973–1990) to 74% (1991–2000) [8]. However, also comparable with our findings, the time period in which the Fontan operation was performed did not impact late survival in those surviving the first year after Fontan completion [8], indicating the improvement to be driven by factors affecting the first postoperative year.

Irrespective of the period, event-free 25-year survival with an extracardiac conduit-type Fontan was 87% and superior to the 79% 25-year survival found in those with a lateral-type Fontan. When comparing 25-year event-free survival conditional of an uneventful first postoperative year, survival was similar between groups, being 90% and 89% for those with an extracardiac and lateral-type Fontan, respectively. While both procedures are superior to the atrio-pulmonary-type Fontan, it is outside the scope of this study to evaluate the superiority of either surgical procedure [8]. While our results indicate improved survival in the extracardiac conduit group, this is likely attributable to the link between type of Fontan and era, and thus differences in both surgical, perioperative, and intensive care management. It should be noted that some institutions have shown excellent early postoperative results with lateral tunnel procedures performed in a later era [20].

Although some centers report excellent HLHS outcomes [7], the 25-year event-free survival in the Danish Fontan population was 61% for the hypoplastic left heart syndrome group compared to 84% among the remaining congenital heart lesions. The difference in survival remained regardless of making comparison conditional to an event-free first year. It is worth noting that the improved survival with an intact Fontan circulation for those operated after 1997 exists despite a greater proportion of the operated individuals having hypoplastic left heart syndrome.

As a secondary aim, we examined the presence of select morbidity and parameters of physical performance in the Danish individuals living with a Fontan circulation. Although based on a non-exhaustive list, the prevalence of morbidity increased with age, affecting 11 out of 61 (18%) in the < 18 age group, 48 out of 126 (38%) in the 18–29 age group, and 35 out of 63 (56%) in the ≥ 30 age group. This compares well to existing studies, where the prevalence is substantial, age-dependent, and driven primarily by the development of arrhythmias [8, 10, 21]. The exercise capacity of the entire Fontan cohort was reduced, with all tested individuals achieving a mean %pred VO2peak of 56%. Although the decline may be slowed or even prevented [17, 22], aligning with most studies, the exercise capacity was inversely related to the age of the group. [17, 23, 24] The mean %pred VO2peak was 65% in the < 18 group, 58% in the 18–29 group, and 51% in the ≥ 30 group. Self-reported activity level is perceived as a predictor of exercise capacity progression and showed an age-related decline in our cohort [17, 22]. Notably, the proportion of individuals unaffected by any morbidity showed a similar decrease with age. Although outside the scope of this study, the relationship between (self-reported) physical activity, progression of exercise capacity, and the potential protective effects with regard to development of Fontan-associated morbidity remains a key area for future research.

### Limitations

This retrospective study, despite being nationwide with complete mortality follow-up, has key limitations. Morbidity data for the Fontan group are incomplete, particularly for individuals undergoing Fontan takedown, cardiac transplant, or who were deceased, as these cases were excluded due to data gaps. Time-to-event analysis was inappropriate due to the retrospective collection of morbidity data from annual follow-up reports, which carried varying margins of error in event timing. The activity cutoff, based on PAQ and QPAQ questionnaires, was created specifically for this study and lacks proper validation, potentially biasing classifications of activity levels based on the age-related choice of questionnaire. Finally, the Danish Fontan population may differ in composition from other countries due to prevalent prenatal screening and high termination rates for fetuses, limiting the generalizability of results.

## Conclusion

In this nationwide follow-up study of the 301 univentricular individuals undergoing Fontan completion in Denmark between 1977 and 2023, we report a 30-year survival of 87% and a freedom from death, transplant, and Fontan takedown of 79%. Fontan completion during or after 1997 or a non-HLHS cardiac diagnosis displayed superior survival, while there was no difference when comparing those who received a lateral-type Fontan with those receiving and extracardiac conduit-type Fontan. For those alive in Denmark with a Fontan circulation, the accumulated prevalence of ventricular dysfunction, atrioventricular valve regurgitation, arrhythmia, and protein-losing enteropathy was 38% and increased with age. The proportion of individuals self-reporting to be active and the percent predicted exercise capacity displayed an inverse decline.

## Supplementary Information

Below is the link to the electronic supplementary material.

Supplementary file1 (XLSX 20 KB)

## Data Availability

The data underlying this article cannot be shared publicly due to data privacy regulations. The data will be shared on reasonable request to the corresponding author and approval from relevant authorities.
